# Vegetarian and vegan diets: benefits and drawbacks

**DOI:** 10.1093/eurheartj/ehad436

**Published:** 2023-07-14

**Authors:** Tian Wang, Andrius Masedunskas, Walter C Willett, Luigi Fontana

**Affiliations:** Charles Perkins Center, University of Sydney, Sydney, NSW, Australia; Faculty of Medicine and Health, University of Sydney, Sydney, NSW, Australia; Charles Perkins Center, University of Sydney, Sydney, NSW, Australia; Faculty of Medicine and Health, University of Sydney, Sydney, NSW, Australia; Department of Epidemiology, Harvard T. H. Chan School of Public Health, Boston, MA, USA; Charles Perkins Center, University of Sydney, Sydney, NSW, Australia; Faculty of Medicine and Health, University of Sydney, Sydney, NSW, Australia; Department of Endocrinology, Royal Prince Alfred Hospital, Sydney, NSW, Australia; Department of Clinical and Experimental Sciences, Brescia University, Brescia, Lombardy, Italy

**Keywords:** Nutrition, Plant-based diets, Vegetarian, Vegan, Cardiovascular disease, Diabetes, Hypertension, Obesity, Dementia, Cancer

## Abstract

Plant-based diets have become increasingly popular thanks to their purported health benefits and more recently for their positive environmental impact. Prospective studies suggest that consuming vegetarian diets is associated with a reduced risk of developing cardiovascular disease (CVD), diabetes, hypertension, dementia, and cancer. Data from randomized clinical trials have confirmed a protective effect of vegetarian diets for the prevention of diabetes and reductions in weight, blood pressure, glycosylated haemoglobin and low-density lipoprotein cholesterol, but to date, no data are available for cardiovascular event rates and cognitive impairment, and there are very limited data for cancer. Moreover, not all plant-based foods are equally healthy. Unhealthy vegetarian diets poor in specific nutrients (vitamin B12, iron, zinc, and calcium) and/or rich in highly processed and refined foods increase morbidity and mortality. Further mechanistic studies are desirable to understand whether the advantages of healthy, minimally processed vegetarian diets represent an all-or-nothing phenomenon and whether consuming primarily plant-based diets containing small quantities of animal products (e.g. pesco-vegetarian or Mediterranean diets) has beneficial, detrimental, or neutral effects on cardiometabolic health outcomes. Further, mechanistic studies are warranted to enhance our understanding about healthy plant-based food patterns and the biological mechanisms linking dietary factors, CVD, and other metabolic diseases.

## Graphical Abstract

**Graphical Abstract:**
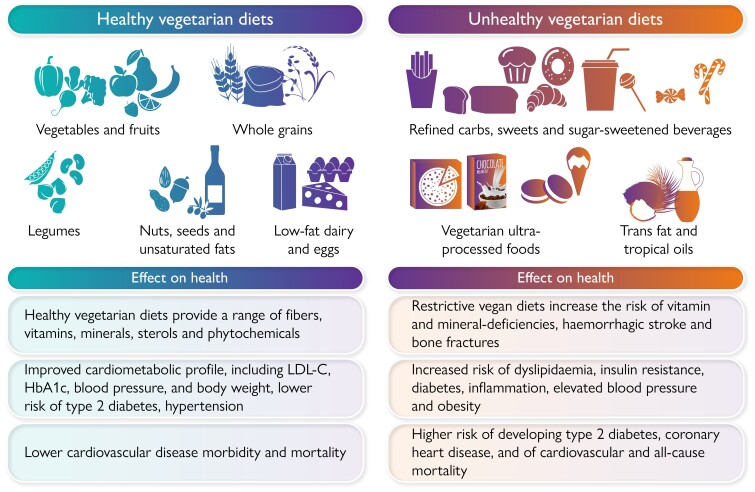
A comparison of healthy vegetarian diets vs. unhealthy vegetarian diets. HbA1c, glycosylated haemoglobin; LDL-C, low-density lipoprotein cholesterol.

## Introduction

Plant-based diets have become increasingly popular thanks to their purported health benefits and more recently for their positive environmental impact.^1^ There are different types of plant-based diets, but in this review, we will focus our attention primarily on vegan (100% plant-based), lacto-ovo vegetarian (i.e. plant-based except for dairy products and/or eggs), and pesco-vegetarian or pescatarian (i.e. plant-based except for fish and seafood with or without eggs and dairy) diets. All vegetarian diets exclude meat (e.g. beef, pork, lamb, venison, chicken, and other fowl) and related meat products.

**Figure 1:**
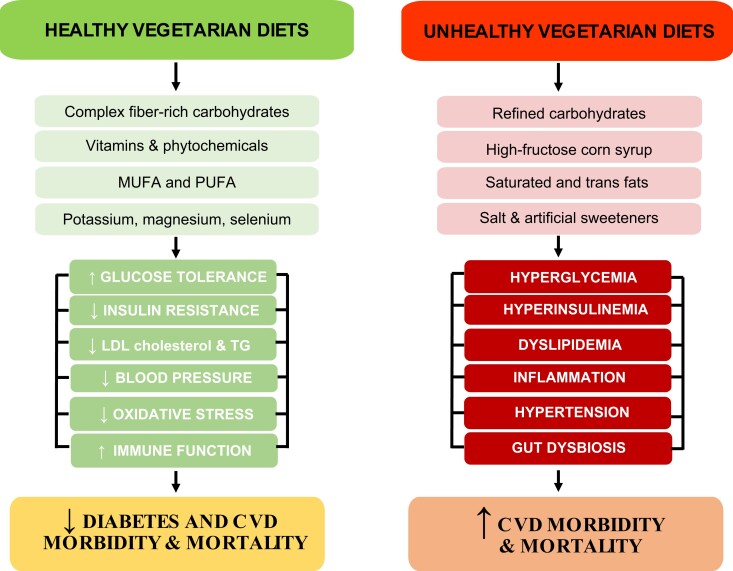
Metabolic effects of healthy and unhealthy vegetarian diets. MUFA, monounsaturated fatty acids; PUFA, polyunsaturated fatty acids; LDL-C, low-density lipoprotein cholesterol; TG, triglycerides.

According to the American and Canadian Dietetic Associations, appropriately planned and supplemented vegan and lacto-ovo vegetarian diets are nutritionally adequate and suitable for individuals in all stages of the life cycle and may provide health benefits in disease prevention and treatment.^2,3^ These statements are supported mainly by cross-sectional and prospective studies with accumulating data from a limited number of clinical randomized trials. Moreover, not all plant-based foods are equally healthy. Vegetarian diets rich in refined flours, hydrogenated oils, high-fructose corn syrup (HFCS), sucrose, artificial sweeteners, salt, and preservatives have been shown to increase morbidity and mortality (*Figure 1*).^4–6^ The purpose of this article is to review succinctly the current knowledge on the effects of vegetarian diets on the risk of developing some of the most common and costly chronic diseases, including cardiovascular disease (CVD), obesity, type 2 diabetes mellitus (T2DM), hypertension, dementia, and cancer, and to discuss what is known about its metabolic and molecular adaptations and effects.

## Evidence acquisition

We searched EMBASE, MEDLINE, CINAHL, Cochrane Central Register of Controlled Trials (CENTRAL), and PubMed, from inception until 20 September 2022. Hand searches of reference lists of reviews, protocols, and clinical trial registries (ClinicalTrials.gov) were performed to supplement searches. Search terms included *diet*, *plant-based*, *vegetarian*, *vegan*, *cardiovascular*, *cardiovascular diseases*, *diabetes*, *T2DM*, *hypertension*, *cancer*, *dementia*, and *cognitive function*. The authors of the ongoing trials were contacted to retrieve preliminary findings and full manuscripts. Both basic science and clinical research studies were reviewed. The published clinical reports that we reviewed included epidemiologic studies, case-control studies, and randomized controlled trials. Quality of data was assessed by taking into account publication in a peer-reviewed journal, number of individuals studied, objectivity of measurements, and techniques used to minimize bias.

## Metabolic and molecular mechanisms associated with vegetarian diets

**Figure 2:**
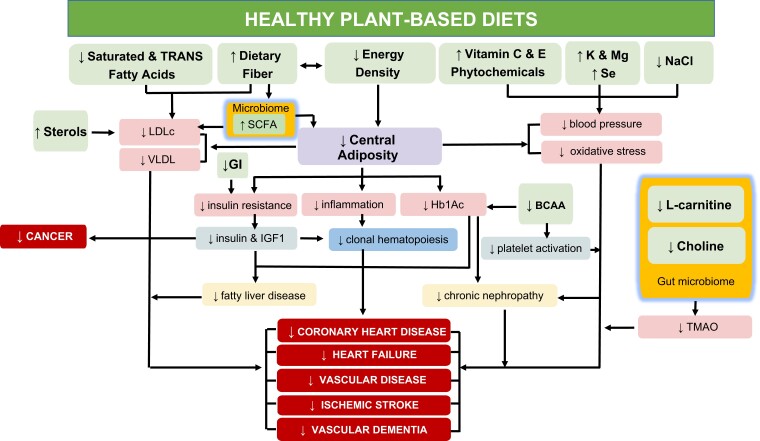
Cardioprotective mechanisms of healthy vegetarian diets. Multiple nutritional effectors of a healthy vegetarian diet modulate important metabolic, hormonal, and immune factors associated with the development of cardio- and cerebrovascular diseases. K, potassium; Mg, magnesium; Se, selenium; NaCl, sodium chloride; SCFA, short-chain fatty acids; LDL-C, low-density lipoprotein cholesterol; VLDL, very-low-density lipoprotein; GI, glycaemic index; BCAA, branched-chain amino acid; TMAO, trimethylamine *N*-oxide.

The precise mechanisms by which well-designed and balanced vegetarian or vegan diets may exert their beneficial effects in lowering the risk of coronary heart disease (CHD) and possibly cancer and dementia are under scrutiny. Many factors have been hypothesized to play a role, including (i) lipid-lowering effect; (ii) glucose-lowering, insulin sensitizing, and hormonal effects; (iii) protection against oxidative stress, inflammation, and hypertension, and (iv) production of intestinal microbial metabolites influencing metabolic and immune health (*Figure 2*).

### Lipid-lowering effect

Several factors can explain why vegetarians have significantly lower levels of plasma cholesterol, especially when they consume minimally processed plant foods. Vegetarians do not consume meat, and vegans also avoid milk, butter, and dairy. Beef, lamb, and pork contain high levels of saturated fat and cholesterol and minimal amounts of polyunsaturated fats; even lean cuts of beef may contain up to 4.5 g of saturated fat per 100 g serving. One-cup serving of whole milk contains 4.5 g of saturated fat, and one tablespoon of butter contains 102 kcal and 7 g of saturated fat. In contrast, one tablespoon of olive oil contains 119 kcal and only 1.9 g of saturated fatty acids. Epidemiological studies have shown a strong linear relationship between saturated fat intake, plasma cholesterol levels, and CHD.^7,8^ Substituting 5% of energy intake from saturated fatty acids with a similar quantity of energy from polyunsaturated fats, monounsaturated fats, or carbohydrates from whole grains is associated with a 25%, 15%, and 9% lower risk of CHD, respectively. However, when saturated fats are replaced with carbohydrates from refined carbohydrates, the risk of developing CHD increases substantially.^9^ Data from randomized clinical trials have demonstrated a cause–effect relationship;^10–12^ replacing saturated fat with vegetable polyunsaturated fats decreases CHD by 30% that is similar to the reduction induced by statin therapy.^13^ Seeds and nuts are excellent sources of polyunsaturated fatty acids and contain soluble and insoluble fibres and sterols that are known to lower cholesterol.^14^ Epidemiological studies suggest that frequent nut consumption can reduce the risk of CHD by 40%–60%.^15^ Data from randomized clinical trials confirm that consuming a diet rich in nuts, viscous fibres from oats, barley, psyllium, and plant sterol ester–enriched margarine can reduce plasma low-density lipoprotein (LDL) cholesterol by 13%.^16^ Moreover, vegetarian diets rich in whole grains, legumes, nuts, and dried fruits can provide ∼15 g of dietary fibre per 1000 kcal. In a 4 month weight loss double-blind, placebo-controlled clinical trial, overweight or obese men and women who received a daily supplement of soluble fibre (3 g *Plantago ovata* husk and 1 g glucomannan) experienced a significant greater drop in LDL cholesterol than those in the placebo group.^17^ Dietary fibres and phytosterols reduce the (re)absorption of cholesterol and bile acids in the small intestine, thus resulting in an increased LDL uptake by the liver.^18,19^ Moreover, foods rich in dietary fibre and with low glycaemic index can lower insulin production and increase the levels of short-chain fatty acids produced by fibre fermentation, which have both been shown to inhibit cholesterol synthesis.^19^

### Glucose-lowering, insulin sensitizing, and hormonal effects

Vegetarians, and especially vegans, tend to have lower body weights than omnivores. In a survey of the American Adventists population, average body mass index (BMI) in omnivores, semi-vegetarians, lacto-ovo vegetarians, and vegans was 28.3, 27.3, 26.1, and 24.1 kg/m^2^, respectively.^20^ Although consuming a vegetarian diet does not require counting calories, results from clinical trials demonstrate that people randomized to a vegetarian diet tend to lose more weight than those consuming Western diets.^21,22^ Preclinical, epidemiological, and clinical studies suggest that distinct dietary interventions may promote atherogenic and metabolic fat depot mobilization differently.^23^ The high-fibre and water content and lower energy density of vegetables, legumes, and whole grains may in part explain this effect. Consumption of diets rich in dietary fibre induces gastric distention, delays gastric emptying, and prevents large fluctuations in postprandial blood glucose.^24^ Short-chain fatty acids produced by the intestinal microbial metabolism of resistant starch and oligosaccharides of minimally refined plant foods induce satiety by inhibiting gastric emptying through incretins such as peptide-YY and glucagon like peptide-1 that markedly reduce blood glucose and body weight in randomized clinical trials.^25–27^ Moreover, whole-food vegan and vegetarian diets may result in fewer bioavailable calories, and it is well known that calorie restriction with adequate nutrition in humans exert a powerful effect in improving glucose tolerance, insulin sensitivity, and many other cardiometabolic, inflammatory, and hormonal factors implicated in the pathogenesis of CVD and cancer.^28–30^ As reviewed elsewhere,^28,31^ excessive (central) adiposity causes insulin resistance, dysregulation of sex hormones and insulin-like growth factor-1 (IGF-1) signalling, low-grade chronic inflammation, and immune dysregulation of natural killer cells and stromal tumour-infiltrating lymphocytes, limiting antitumour responses. Compensatory hyperinsulinaemia together with increased bioavailability of oestradiol, testosterone, and IGF-1 promotes cell proliferation and genomic instability through activation of the PI3K/AKT and p66^shc^ pathways, which have been associated with increased risk of multiple cancers, including breast, endometrial, prostate, and colon cancer.^28,31^

Additional mechanisms mediating the insulin sensitizing and glucose-lowering effects of healthful minimally processed vegetarian diets are the low glycaemic index/load and the lower intake of protein, especially of sulphur and branched-chain amino acids. Estimated daily protein intake for omnivores in Western societies is ∼90–100 g of which ∼70%–85% is animal proteins rich in methionine, valine, leucine, and isoleucine. Results from both population and randomized experimental diet interventions show that high protein intake, especially of branched-chain amino acids, is associated with an increased prevalence and risk of developing pre-diabetes and T2DM.^32^ Diabetes risk increases by 20%–40% for every 10 g of protein consumed in excess of 64 g per day.^33,34^ Interestingly, in some studies, high intake of animal protein, but not of plant protein, was associated with the higher risk of developing T2DM.^32,34,35^ In weight loss trials of obese women, high protein intake (1.3 g kg^−1^ per day including two servings of a whey protein isolate) completely prevented the markedly improved insulin sensitivity observed in women consuming a normal protein diet (0.8 g kg^−1^ per day) who lost the same amount of body weight and visceral and liver fat.^36^ Furthermore, dietary branched-chain amino acid (BCAA) restriction in mice recapitulates many of the beneficial effects of protein restriction observed in rodents and humans, including reduced adiposity, increased glucose tolerance, and increased energy expenditure, but not increased FGF21 levels.^37^ In contrast, high dietary intake of BCAA increases platelet activation and arterial thrombosis risk by enhancing tropomodulin-3 propionylation.^38^ Consistently, data from two trials demonstrated that consuming high-protein diets (comprising dairy and meat products and whey protein supplements) cause a reduction in insulin sensitivity and an associated increase in blood insulin levels.^39,40^ In an another trial of patients with T2DM, high consumption of chicken, fish, eggs, low-fat milk, and cheeses prevented the expected improvements in glucose metabolism and insulin sensitivity induced by a 2 month weight loss intervention.^41^ High-protein diets, particularly those rich in leucine, can also play a role in promoting atherosclerosis and plaque instability in mice by exacerbating macrophage apoptosis induced by atherogenic lipids, via mTORC1-dependent inhibition of mitophagy and accumulation of dysfunctional mitochondria.^42^

### Protection against oxidative stress, inflammation, and hypertension

Well-designed vegetarian diets rich in vegetables, whole grains, legumes, nuts, seeds, and fruits provide a wide range of vitamins (vitamin C, vitamin E, and beta-carotene), minerals (selenium), and phytochemicals (tannins, phenols, alkaloids, and flavonoids) with xenohormetic effects.^43^ Numerous large observational studies suggest that an inverse relationship exists between antioxidant and polyphenol intake and the risk of developing diabetes, CVDs, cancer, and possibly dementia.^44^ High intake of dietary antioxidants and phytochemicals may reduce the risk of developing atherosclerotic plaques because it triggers adaptive modulations of stress-response enzymes and receptors that prevent lipoprotein oxidation, endothelial dysfunction, and immune activation.^45,46^ Findings from large prospective studies suggest that dietary patterns with higher inflammatory potential are significantly associated with higher level of systemic and vascular inflammation, an unfavourable lipid profile, and ultimately with a higher incidence of CHD and stroke.^47^ Dietary patterns with lower inflammatory potential are those that favour foods rich in dietary antioxidants and vegetable fibre (e.g. green leafy and dark yellow vegetables, whole grains, fruit, tea, and coffee) and avoid red and processed meat and refined liquid and solid carbohydrates.^48–52^

Diets rich in vegetable fibre, potassium, and magnesium and low in sodium, especially when associated with a healthy body weight and regular endurance exercise training, markedly lower systolic and diastolic blood pressure,^53–56^ which is a powerful risk factor for the development of CHD, heart failure, stroke (both ischaemic and haemorrhagic), and dementia. Indeed, data from epidemiological and genetic causal inference studies show that elevated systolic blood pressure, insulin resistance, and excess adiposity at midlife are important risk factors for developing cognitive impairment and Alzheimer’s disease because they cause endothelial dysfunction and vascular damage to the brain, particularly at the level of perforating cerebral arteries and neurovascular units.^57,58^ In contrast, reduction of systolic blood pressure prevents and/or slows progression of cognitive impairment to dementia.^59^

### Modulation of gut microbiome function and effect on human metabolic state

Diet composition has a pervasive effect in modulating systemic microbiome biology. Metagenomic data show that specific nutrients, especially insoluble fibre, and protein intake deeply influence gut microbiota structure and function and the production of a growing list of metabolically active molecules.^60,61^ For instance, unlike vegetarians diets, Western diets rich in red meat, eggs, and cheese contain higher concentrations of nutrients such as choline and L-carnitine that increase the microbial production of trimethylamine *N*-oxide (TMAO).^62,63^ Animal and human studies have shown that higher levels of circulating TMAO increase the risk of developing CVD, independent of traditional cardiometabolic risk factors, by inducing vascular inflammation and platelet activation.^64,65^ In contrast, healthful plant-based diets rich in whole grains, legumes, and nuts can markedly increase the intake of dietary fibres, key fermentable substrates for the proliferation of Bacteroidetes and the production of short-chain fatty acids such as acetate, propionate, and butyrate.^66,67^ Experimental animal data indicate that these microbial metabolites exert powerful blood pressure–lowering and immune-modulating effects, via activation of specific G-protein–coupled receptors expressed on enteroendocrine and intestinal immune cells.^61,68^ Long-term consumption of vegetarian diets has also been associated with more phylogenetic biodiversity of stool microbiota; in contrast, multigenerational exposure to Western diets poor in ‘microbiota-accessible carbohydrates’ causes an extinction of specific bacterial lineages, which impairs immune function and maturation, and increases the risk of developing a range of metabolic, inflammatory, allergic, and autoimmune diseases.^69,70^ Interestingly, data from the DIRECT-PLUS trial show that a calorie-restricted and (almost) red-meat-free version of the Mediterranean diet enriched in plant-based proteins (Green-MED diet) is superior to the classical Mediterranean diet in improving the 10-year Framingham risk score and in lowering waist circumference, intrahepatic fat, LDL cholesterol, diastolic blood pressure, C-reactive protein, and HOMA insulin resistance.^71^ These cardiometabolic beneficial effects were partially mediated by a major shift in the composition and function of the gut microbiome, including enrichments in the genus Prevotella and reductions in the genus Bifidobacterium with associated inhibition in BCAA biosynthesis and up-regulation of BCAA degradation enzymatic pathways.^72^ This is crucial because a growing body of evidence show that reprogramming microbial functions through long-term adherence to healthier plant-rich diets has profound effects in shaping physiologic response to specific nutrients, to calorie restriction, and to other features of host biology that are instrumental in promoting health and longevity.^73,74^

## Evidence from prospective studies

Prospective epidemiological studies have suggested that consuming vegetarian diets might have protective effects against the development of obesity, diabetes, hypertension, CHD, several type of cancers, and, most recently, cognitive decline. Whether these associations are causal deserves careful consideration of all available evidence, including data from other types of studies.

### Hypertension

Findings from observational studies suggest that people consuming vegetarian and vegan diets have lower blood pressure than people eating Western diets, even after adjusting for age, sex, and BMI.^75^ Compared with Seventh-day Adventist who are omnivores, those who follow a vegetarian diet have lower blood pressure and a reduced incidence of hypertension, independent of body weight and sodium intake.^76^ Data from multiple observational studies including three large prospective American cohort studies suggest that consuming red meat and poultry is associated with an increased risk of hypertension, independent of vegetable, whole grain, and fruit intake.^77^

#### Type 2 diabetes mellitus

Several studies suggest protective effects of vegetarian diets in the prevention of T2DM. Findings from the Adventist Health Study-2 (41 387 participants free of diabetes followed for 2 years) found that, even after controlling for multiple confounding factors, vegetarians had a significantly lower risk of T2DM than omnivores.^78^ The most apparent protective effect was for vegan diets with a 62% risk reduction, followed by semi-vegetarian (51% reduction) and lacto-ovo vegetarian (38% reduction) diets. The Adventist Mortality Study and Adventist Health Study followed a cohort of 8401 individuals for more than 17 years.^79^ After controlling for weight and weight change, long-term adherence to a diet incorporating weekly meat intake was associated with a 38% higher risk of T2DM compared with a vegetarian diet with no meat intake. This finding are supported by data from a joint analysis of three large cohort studies (the Health Professionals Follow-up Study, *n* = 26,357; the Nurses’ Health Study, *n* = 48,709; and the Nurses’ Health Study II, *n* = 74,077) confirming a statistically significant association between red meat consumption and an increased risk of T2DM (*P* < .001 for all studies).^80^ After adjusting for initial BMI and concurrent weight gain, a daily increase of > 0.5 servings of red meat was linked with a 30% higher risk of T2DM. In contrast, reducing red meat intake by > 0.5 servings/day was associated with a 14% lower risk of T2DM.

#### Cardiovascular disease

A joint analysis of five prospective studies including 76 172 individuals has shown a lower CHD mortality in vegetarians than in omnivores: 34% less in lacto-ovo vegetarians and pesco-vegetarians and 26% lower in vegans.^81^ Another meta-analysis of 7 studies (124 706 participants) report a 29% decreased mortality from CHD in vegetarians than omnivores.^82^ The EPIC-Oxford cohort study (44 561 participants) showed a 32% risk reduction of CHD in vegetarians than non-vegetarians.^83^ However, subsequent studies suggest that the protective effect against CHD of vegetarian diets seems to be almost exclusively limited to the Seventh-day Adventists, who don’t smoke, don’t drink alcohol, do regular physical activity, and are very religious and socially connected.^84^ Indeed, data from epidemiological studies of English and German vegetarians show only a modest protective effect against cardiovascular and overall mortality.^85–87^ A German prospective study of 1225 vegetarians and 679 health-conscious non-vegetarians has shown that there is no difference in mortality among vegetarians and this control group of health-conscious individuals consuming meat three to four times per month.^88^ Cigarette smoking, obesity, alcohol intake, and exercise patterns seem to explain most of the differences in cardiovascular mortality among these different groups. Another potential problem is diet quality, which can vary greatly among both vegetarian and non-vegetarians.^4,5,89^

The effects of vegetarian diets on major cardiometabolic risk factors (i.e. hypercholesterolaemia, dyslipidaemia, hypertension, T2DM, and obesity) are more consistent. Well-educated vegetarians who consume balanced diets tend to have a lower body weight than non-vegetarians^21^ together with lower levels of cholesterol, glucose, and blood pressure.^90^ A recent umbrella review integrated evidence from 20 meta-analyses and found that people following vegetarian diets had significantly lower total cholesterol and LDL cholesterol than people consuming Western diets.^91^ On average, total and HDL cholesterol are ∼0.36 and 0.10 mmol/L, respectively, lower in vegetarians than in omnivores.^92^

#### Cancer

A meta-analysis of 7 epidemiological studies (124 706 participants) found an 18% lower cancer incidence in vegetarians than omnivores {relative risk [RR]: 0.82 [95% confidence interval (CI): 0.67, 0.97]}.^82^ Results from the EPIC-Oxford study on a cohort of 65 000 men and women found that the overall cancer risk was 10% lower in vegetarians and 18% lower in vegans than in meat-eaters.^93^ However, after correcting for multiple confounding factors, only stomach and haematological cancers were significantly lower, while cervical cancer was 90% higher in vegetarians. Recent data from the UK Biobank prospective study on 409 110 participants show that compared with omnivores, vegetarians had a 13% and pescatarians a 7% lower overall cancer risk, respectively. In this study, vegetarians had a lower risk of colorectal and prostate cancer, and pescatarians had a lower risk of melanoma. However, when these data were pooled with eight previously published studies in a meta-analysis, only the association with colorectal cancer persisted.^94^ These findings suggest that other factors beyond vegetarian diets may explain these associations. The incidence of lung cancer, for example, is lower in vegetarians than in people consuming typical Western diets, but this seems due primarily to the reduced smoking habit of vegetarians. No difference has been reported for lung cancer risk for vegetarians in maximally adjusted models.^95–97^ The incidence of colon cancer is reduced by 22% among Seventh-day Adventist vegetarians, but not in British vegetarians. In the latter group, for example, it seems that vegans have an even higher risk of colon cancer, while in pesco-vegetarians, there is a 33% reduction, even after correcting for body weight.^95^ The quality of diet probably plays a major role. Indeed, unhealthy plant-based diets rich in refined and processed carbohydrates and unhealthy fats are associated with higher risk of colon cancer, but healthy plant-based diets enriched in whole grains, legumes, and vegetables are associated with lower incidence of colorectal cancer, especially KRAS-wildtype subtype.^6^ The risk of developing breast cancer is no different between vegetarian and non-vegetarian women in most studies, and some epidemiological data in Adventist and British women suggest vegans, but not lacto-ovo vegetarians, may have an increased risk.^98^ The same is true for prostate cancer, with the risk no different among lacto-ovo vegetarians and omnivores but 34% lower in the Adventists vegans.^99^ A lower intake of dairy products may explain this association because milk consumption increases serum IGF-1 levels, a risk factor for prostate cancer, breast, and colon cancer.^100^

#### Dementia

Very little is known about the effects of vegetarian diets on cognitive function and dementia risk. A recent systematic review and meta-analysis suggests that vegetarian diets are not associated with any significant improvement in memory when compared with omnivorous diets, but heterogeneity among studies was very high.^101^ Findings from a small prospective study (5710 participants with 121 incident cases) conducted in Taiwan suggest that vegetarians might have a lower risk of dementia than non-vegetarians.^102^

## Evidence from randomized clinical trials

### Hypertension

Data from a meta-analysis of 7 clinical trials including 311 participants show that consuming a vegetarian diet is associated with a reduction of mean systolic [−4.8 mmHg (−6.6 to −3.1)] and diastolic [−2.2 mmHg (−3.5 to −1.0)] blood pressure compared with non-vegetarian diets.^103^ A meta-analysis of 11 trials and 983 participants showed that strict plant-based (vegan) diets seem less effective than less restrictive diets and reduced systolic [−4.10 mmHg (−8.14 to −0.06)] and diastolic [−4.01 mmHg (−5.97 to −2.05)] blood pressure only in patients with a baseline systolic blood pressure (SBP) ≥130 mmHg.^104^ A recent meta-analysis of randomized trials show that the lacto-ovo vegetarian diet is as effective as other healthy diets containing some animal products [Dietary Approaches to Stop Hypertension (DASH) and healthy Nordic diet] at reducing blood pressure. In contrast, vegan diets did not significantly reduce blood pressure unless caloric restrictions was also prescribed,^105^ suggesting that complete elimination of animal food is not required for lowering blood pressure and might even increase haemorrhagic stroke risk, possibly due to very low intake of saturated fat.^93^ Other factors such as calorie restriction and weight loss,^30,54,106^ lower dietary sodium and high potassium and magnesium intake,^53,55^ and regular endurance exercise training^56^ are important factors beyond fibre-rich plant food consumption. Moreover, findings from a meta-analysis of 15 randomized trials show that reduced alcohol consumption dose-dependently lowers systolic and blood pressure in both in non-hypertensive and hypertensive individuals.^107^

### Type 2 diabetes mellitus

The results of a recent meta-analysis of nine randomized clinical trials provide evidence that vegetarian diets can significantly reduce fasting glucose (range 0.1–1.0 mmol/L) and glycosylated haemoglobin (HbA1c) (range 0.12%–0.45%) together with LDL cholesterol (range 0.04–0.2 mmol/L) and body weight (range 1.3–3.0 kg) in T2DM patients.^108^ Interestingly, one randomized clinical trial comparing a low-fat vegan diet with the American Diabetes Association (ADA) diet demonstrated that both diets caused significant improvements in HbA1c, body weight, plasma lipid concentrations, and urinary albumin excretion in individuals with T2DM.^109^ Forty-three percent of patients randomized to the vegan group and 26% of those allocated to the ADA group reduced the use of glucose-lowering drugs. Moreover, among medication-stable patients, the effects of the low-fat vegan diet on HbA1c, weight, waist circumference, and LDL cholesterol were significantly greater than in the control group. Similar improvements in HbA1c levels have been found in a population of Korean men and women affected by T2DM.^110^ Thus, these trials suggest that low-fat vegan diets might be more effective than conventional diabetic diets in glycaemic control, but more studies with long-term follow-up are needed to confirm these findings. *Table 1* summarizes the ongoing clinical trials with vegetarian diet interventions in people with T2DM.

**Table 1:** Study characteristics of completed and ongoing clinical trials in people with type 2 diabetes mellitus

Author, year, country	Study design; intervention duration	Registration number; status	Study arms, sample size	Population condition, gender, mean age (years)	Diet description		Relevant biomarkers [MD (95% CI)]
					Intervention	Comparison	
Barnard *et al.*, 2006,^109^ USA	22 weeks, randomized, controlled trial, parallel arm; 52 weeks follow-up	NCT00276939; completed trial	(1) The LF vegan Diet (49) (2) The ADA diet (50)	T2DM and being overweight (BMI ≥ 25 kg/m^2^); gender: (1) 27 F, 22 M; (2) 33 F, 17 M; mean age (range): (1) 57 (35–82); (2) 55 (27–80)	The LF, vegan diet: no animal products and added fats; consisted of F & V, grains, and legumes, favour low-GI foods; vitamin B12 supplement (100 mcg) taken every other day	The ADA diet: participants (with BMI >25 kg/m^2^) were prescribed 500–1000 kcal energy deficits; vitamin B12 supplement (100 mcg) to be taken every other day	LDL: −0.02 [−0.31, 0.27] mmol/L; HbA1c: −0.40 [−0.85, 0.05]%; SBP: −0.2 [−5.4; 5.0] mmHg
Barnard *et al.*, 2018,^111^ USA	20 weeks, randomized, controlled trial, parallel arm	NCT01222429; completed trial	(1) The LF vegan diet (22) (2) The portion-controlled eating plan (23)	T2DM (HbA1c 6.5%–10.5%) and overweight (BMI ≥ 25 kg/m^2^); gender: (1) 13 F, 9 M; (2) 11 F, 12 M; mean (range): (1) 62 (41–79); (2) 61 (30–75)	The LF, low-GI, vegan diet: consisted of whole grains, F & V, and legumes; no animal products and added oils; no restrictions on energy or carbohydrate intake	The portion-controlled eating plan: energy limits when needed for weight loss (calorie-restricted −500 kcal/d) and guidance on portion sizes	LDL: 0.06 [−0.01, 0.13] mmol/L; SBP: 5.5 [2.7, 8.3] mmHg

Author, year, country	Study design; Intervention duration	Registration number; status	Study arms, sample size	Population condition, gender, mean age (years)	Diet description		Relevant biomarkers (MD[95%CI])
					Intervention	Comparison	
Bunner *et al.*, 2015,^112^ USA	20 weeks randomized, parallel arm, controlled trial	NCT01690962; completed trial	(1) The LF vegan diet + B12 supplement (17) (2) No intervention + B12 supplement (17)	T2DM with painful diabetic neuropathy for ≥6 months; gender: (1) 11 F, 6 M; (2) 8 F, 9 M; mean age: (1) 57 ± 6; (2) 58 ± 6	The LF, vegan diet first: no animal products; focused on grains, F & V, and legumes; limited fat to 20–30 g/d; favoured low-GI foods; daily vitamin B12 (1000 mcg)	No dietary change except for daily vitamin B12 supplement (1000 mcg)	LDL: −0.21 [−0.65, 0.23] mmol/L; HbA1c: −0.80 [−1.51, −0.09] %; SBP: −7.2 [−20.1, 5.7] mmHg
Kahleova *et al.*, 2011,^113^ Czech Republic	24 weeks RCT, open, parallel arm. The second 12 weeks were combined with aerobic exercise.	NCT00883038; completed trial	(1) The lacto-vegetarian diet (37) (2) Conventional diabetic diet (37)	T2DM (HbA1c 6%–11%) and being overweight (BMI 25–53 kg/m^2^); gender: (1) 20 F, 17 M; (2) 19 F, 18 M; mean age (years): (1) 55 ± 7.8; (2) 58 ± 4.9	The lacto-vegetarian diet: consisted of grains, F & V, and legumes; animal products limited to ≤1 portion of LF yogurt/d; vegetarian meals provided in vegetarian restaurants; calorie-restricted (−500 kcal/d); daily vitamin B12 supplement (50 mcg)	The conventional diabetic diet: follow dietary guidelines of the DNSG or the EASD; meals provided; calorie-restricted (−500 kcal/d); daily vitamin B12 supplement (50 mcg)	LDL: −0.04 [−0.28, 0.20] mmol/L; HbA1c; −0.09 [−0.49, 0.31]%

Author, year, country	Study design; Intervention duration	Registration number; status	Study arms, sample size	Population condition, gender, mean age (years)	Diet description		Relevant biomarkers (MD[95%CI])
					Intervention	Comparison	
Lee *et al.*, 2016,^114^ Korea	12 weeks, randomized, controlled trial, parallel arm	CRiS KCT0001771; completed trial	(1) The vegan diet + brown rice (53) (2) The conventional diabetic diet (53)	T2DM (HbA1c 6.0%–11.0%); gender: (1) 40 F, 6 M; (2) 35 F, 12 M; mean age: (1) 57.5 ± 7.7 (2) 58.3 ± 7.0	The vegan diet + brown rice: consisted of F & V, whole grains, and legumes; no animal products, white rice, and processed food made of rice/wheat flour; have brown rice; favour low-GI foods; energy intake, and portion sizes not restricted	The conventional diabetic diet: followed the treatment guidelines by the Korean Diabetes Association (2011); restrict individualized daily energy intake based on weight, physical activity, need for weight control, and compliance	LDL: −0.04 [−0.29, 0.21] mmol/L; HbA1c: −0.30 [−0.61, 0.01] %; SBP: 2.5 [−4.4, 9.4] mmHg
Nicholson *et al.*, 1999,^115^ USA	12 weeks randomized controlled trial, parallel arm	Completed trial	(1) The LF vegan diet (7) (2) The conventional LF diet (6)	T2DM; gender: (1) 3 F, 4 M; (2) 2 F, 2 M; mean (range): (1) 51 (34–62); (2) 60 (51–74)	The LF vegan diet: no animal products, added oils, and refined carbohydrates; consisted of F & V, whole grains, and legumes; the diet was adequate in all nutrients except vitamin B12	The LF diet: emphasized fish and poultry, rather than red meat	HbA1c: −0.40 [−2.00, 1.20] %; SBP: 8.5 [−9.1, 26.1] mmHg

Author, year, country	Study design; Intervention duration	Study name/registration number; status	Study arms, sample size	Population condition, gender, mean age (years)	Diet description	
					Intervention	Comparison
Campbell *et al.* 2018,^116^ USA	RCT, crossover design, each for 2 weeks with a 4–6 week washout	NCT 05414851; not yet recruiting	(1) WFPB diet (20) (2) Low-carbohydrate diet (20)	Aged >50y with diagnosis of: hypertension or hyperlipidaemia or pre-diabetes or diabetes or obesity	The WFPB diet: exclude animal foods, oils, and solid fats	The low-carbohydrate diet: ‘nut’ carbs <50 g/d
Kahleova *et al.*, 2019,^117^ USA	22 weeks, RCT, crossover design	NCT04088981; not yet recruiting	(1) The LF vegan diet (30) (2) The portion-controlled diet (30)	Individuals aged ≥ 18 years with T2DM and BMI 26–40 kg/m^2^	The LF, vegan diet: no animal products and added oils; no restriction on energy intake	The portion-controlled diet: an individualized diet; reduce 500 kcal/d for overweight participants
Sangeetha *et al.*, 2018, India	Randomized, parallel arm trial	CTRI/2018/10/015896; NI on recruitment	(1) Vegetarian diet (2) Mixed diet	T2DM	Vegetarian diet: consume the prescribed vegan, pesco-, and ovo-lacto-vegetarian diet	Diabetics on mixed diet: the prescribed non-vegetarian diet
Wright *et al.*, 2017,^118^ New Zealand	10 weeks RCT, parallel arm; 42 weeks follow-up	ACTRN12617000541303; results not published yet	(1) WFPB diet (24) (2) Usual care (24)	Obese (BMI ≥ 30 kg/m^2^) and T2DM/pre-diabetes	WFPB approach: avoid animal products; no energy restriction; daily 50 mcg vitamin B12 supplement	Usual care

ADA, American Diabetes Association; CI, confidence interval; F & V, fruits and vegetables; HbA1c, haemoglobin A1c; LDL-C, low-density lipoprotein cholesterol; LF, low-fat; LOV, lacto-ovo vegetarian diet; MD, mean differences; SBP, systolic blood pressure; DNSG, Diabetes and Nutrition Study Group; EASD, European Association for the Study of Diabetes; NA, not applicable; RR, relative risk; F, females; M, males; NI, no information; OR, odds ratio; RCT, randomized controlled trial; IQR, interquartile range; M, males; WFPB, whole foods plant-based.

### Cardiovascular disease

Randomized clinical trials are usually considered gold standard studies for evaluating the cause–effect relationship of health interventions, although misleading conclusions can easily occur due to low adherence to the intervention or inadequate follow-up time. To the best of our knowledge, there are no randomized clinical trials that have tested the effects of vegetarian diets alone on CHD event rates. The Lifestyle Heart Trial was designed to investigate the effects of an intensive lifestyle programme comprising a 10% fat whole foods vegetarian diet together with aerobic exercise, stress management training, smoking cessation, and group psychosocial support in 48 patients with moderate to severe CHD.^119^ Only 20 of the 28 patients randomized to the experimental group completed the 5-year follow-up and experienced a small but significant regression of coronary atherosclerosis (a 7.9% relative improvement) and a decrease in symptomatic and scintigraphic myocardial ischaemia.^119,120^ In contrast, patients randomized to the usual care control group who completed the study (*n* = 15) experienced a 27.7% relative worsening of the average percent diameter stenosis. However, this was a very small, under-powered study that does not allow to differentiate the effects of the vegetarian regimen from those induced by the very low-fat diet, regular aerobic exercise, smoking cessation, and stress reduction programme.

Many randomized clinical trials have tested the effects of different forms of vegetarian diets on cardiometabolic risk factors. Recent meta-analyses reported that vegetarian diets significantly improve several risk factors, including body weight (1.2–2.8 kg reduction),^121^ SBP (3.3–7.6 mmHg reduction),^103,105^ total cholesterol (0.32–0.76 mmol/L reduction), LDL cholesterol (0.32–0.59 mmol/L reduction), high-density lipoprotein (HDL) cholesterol (0.088–0.093 mmol/L reduction),^122^ and HbA1c (0.15%–0.65% reduction).^123^ A crossover randomized trial showed that a vegetarian diet was as effective as the Mediterranean diet in reducing body weight and fat mass, but the former resulted in significantly lower LDL cholesterol levels in middle-aged men and women.^22^ However, many of these meta-analyses were focused on relatively healthy populations or did not stratify patients for gender and disease status. Evidence of the metabolic effects of plant-based diets in people with CVD is limited. *Table 2* summarizes the ongoing clinical trials with vegetarian diet interventions in people with CVD.

**Table 2:** Study characteristics of completed and ongoing clinical trials in people with cardiovascular diseases

Author, year, country	Study design; intervention duration	Study name/registration number; status	Study arms, sample size	Population condition, gender, mean age (years)	Diet description		Relevant biomarkers [MD (95%CI)]
					The vegetarian diet	Comparison	
Aldana *et al.*, 2007,^124^ USA	1 year RCT, parallel arm	Completed trial	(1) The Dr. Ornish Programme (46) (2) Traditional cardiac rehabilitation (47)	CHD; males: (1) 47.8%; (2) 64.6%; mean age: (1) 60.9 ± 9.7; (2) 62.2 ± 8.9	The very LF, LOV diet: no animal proteins except for non-fat dairy and egg whites; liberal consumption of F & V, whole grains, and legumes; one serve of soy food/day; a multivitamin and a flax source of omega-3-fatty acids	No dietary intervention: traditional cardiac rehabilitation	LDL-C: −0.07 (−0.39, 0.25) mmol/L; SBP: 0.6 (−6.8, 7.9) mmHg
Ornish *et al.*, 1990,^119,125^ USA	Initially 1 year RCT, but extended the study for an additional 4 years, parallel arm	Completed trial	(1) The LF LOV diet (53) (2) Usual care (40)	CHD; gender: (1) 1 F, 21 M; (2) 4 F, 15 M; mean age: (1) 56.1 ± 7.5; (2) 59.8 ± 9.1	The LF (10% energy from fat), LOV diet: consisted of F & V, grains, legumes, and soybean products; no animal proteins except for non-fat dairy and egg whites; vitamin B12 supplemented; no calorie restriction; salt restricted for hypertensive patients; caffeine eliminated, alcohol limited to ≤2 units/d	Usual care: not asked to make lifestyle changes	SBP: 2.0 (−10.2, 14.2) mmHg

Author, year, country	Study design; Intervention duration	Study name/registration number; status	Study arms, sample size	Population condition, gender, mean age (years)	Diet description		Relevant biomarkers (MD[95%CI])
					The vegetarian diet	Comparison	
Shah *et al.*, 2018,^126,127^ USA	8 weeks RCT, open-label, blinded, parallel arm	NCT02135939; completed trial	(1) The vegan diet (50) (2) The AHA diet (50)	CHD: > 3/4 had dyslipidaemia. gender: (1) 57 F, 43 M; (2) 58 F, 42 M; median age (IQR): (1) 63.0 (57.0–68.0); (2) 59.5 (53.0–67.0)	The vegan diet: whole-food plant-based diet with no processed foods; no animal products; vitamin B12–fortified soy milk; given the cookbooks *Simply Vegan* (Baltimore: Vegetarian Resource Group, 2012)	The AHA diet: given the AHA LF, *Low-Cholesterol Cookbook* (New York: Clarkson Potter, 2008)	LDL-C: −0.09 (−0.27, 0.09) mmol/L; HbA1c: 0.00 (−0.13, 0.13) %
Toobert *et al*., 2000,^128^ USA	2 years, RCT, parallel arm	Completed trial	(1) Prime Time programme: very LF, LOV diet (17) (2) Usual care (11)	Women with CHD; mean age: (1) 64 ± 10; (2) 63 ± 11	The reversal diet (very LF, LOV diet): no animal products other than egg whites and non-fat yogurt; no added oils or other concentrated fats; < 10% calories from fat	Usual care	LDL-C: −0.12 (−0.72, 0.48) mmol/L; SBP: −9.0 (−27.7, 9.7) mmHg
Cassidy *et al.*, 2022,^129^ Australia	1 year, RCT, parallel arm	ACTRN12620001151921; recruiting	(1) Intensive lifestyle programme (75) (2) AHA diet (75)	Individuals with CHD aged 18–80 years	The 5:2 pesco-vegetarian diet: no animal foods other than fish, egg, and dairy products; 2 non-consecutive fasting days per week	The AHA diet: general dietary guidelines from the AHA	NA

CI, confidence interval; CHD, coronary heart disease; CR, calorie-restricted; F & V, fruits and vegetables; LDL-C, low-density lipoprotein cholesterol; LF, low-fat; LOV, lacto-ovo vegetarian diet; MD, mean differences; RCT, randomized controlled trial; SBP, systolic blood pressure; AHA, American Heart Association; HbA1c, haemoglobin A1c; IQR, interquartile range; NA, not applicable.

### Cancer

To our knowledge, only one randomized clinical trial to date has investigated the effects of a vegan diet on cancer outcomes, and preliminary data show a significant reduction in body weight and cholesterol at 8 weeks.^130^  *Table 3* summarizes the ongoing interventional clinical trials on the effects of vegetarian diets in people with cancer.

**Table 3:** Study characteristics of clinical trials in people with cancer

Author, year, country	Study design; intervention duration	Registration number; status	Study arms, sample size	Population condition, gender, mean age (years)	Diet description		Relevant biomarkers (MD)
					The vegetarian diet	Comparison	
Campbell *et al.*, 2022,^130^ USA	Randomized trial, parallel arms; 8 weeks	NCT03045289	(1) WFPBD + multivitamin (19) (2) Usual diet + multivitamin (9)	Women age ≥18 years with metastatic breast cancer	A WHPBD: provide 3 meals/d; no animal products, refined flours; encourage fruits and vegetables	A usual diet + daily multivitamin	Final value (adjusted for baseline): weight (kg): (1) 73; (2) 77; *P* < .01 LDL-C (mmol/L): (1) 2.1; (2) 2.76; *P* < .01 SBP (mmHg): (1) 108.5; (2) 114.8; *P* = .06 CA 27.29 (U/mL): (1) 24.4; (2) 27.7; *P* = .09
Gulley *et al.*, 2021,^131^ USA	RCT, parallel arms; 16 weeks	NCT04866810; recruiting	(1) High-fibre PBD + exercise prescription (40) (2) Standard diet + exercise guidelines (40)	Age ≥18 years with histologically or cytologically confirmed unresectable melanoma	A high-fibre PBD: ideally a vegan diet, provided a sample meal plan and vegan recipes; ≥30 g fibre/d	Standard diet from healthy eating guidelines	NA

Author, year, country	Study design; Intervention duration	Registration number; status	Study arms, sample size	Population condition, gender, mean age (years)	Diet description	
					The vegetarian diet	Comparison
Jeppsen *et al*., 2022,^132^ USA, India	Randomized trial, parallel arms; 12 weeks	NCT05410002; active, not recruiting	(1) Vegetarian + N-111 (2) Non-vegetarian + N-111 (3) Vegetarian + placebo (4) Non-vegetarian + placebo (5) Vegetarian control (6) Non-vegetarian control	Age ≥ 25, < 80 years with positive diagnosis of breast or prostate cancer	Vegetarian diet	Usual diet
Iyengar *et al.*, 2020,^133^ USA	Randomized trial, parallel arms; 8 weeks	NCT04298086; recruiting	(1) CR vegetarian diet (31) (2) Nutrition + physical activity counselling (31)	Overweight post-menopausal women with breast cancer	A vegetarian diet: provide lunch + dinner for 6 d/week; no animal products; structured exercise	A home-based, physical activity programme and nutrition counselling
Avivi *et al.*, 2021,^134^ Israel	Single-arm clinical trial; 3 years	NCT04957693; recruiting	Vegan diet and lifestyle changes (40)	Age ≥18 years with diagnosis of indolent lymphoma	Specifically designed vegan nutrition, physical activity (mostly aerobic), and stress reduction	Not applicable
Shah *et al.*, 2021,^135^ USA	Single-arm clinical trial; 24 weeks	NCT04920084; recruiting	WFPBD (20)	Age ≥18 years with smoldering multiple myeloma or MGUS	A WHPBD: provide lunch + dinner for 6 d/week; no/minimal animal products; access to a coach daily	Not applicable

LDL-C, low-density lipoprotein cholesterol; MD, mean differences; NA, not applicable; SBP, systolic blood pressure; WFPBD, whole-food plant-based diet; CR, calorie-restricted; MGUS, monoclonal gammopathy of undetermined significance; N-111, nutraceutical supplement, ingredients unspecified.

### Dementia

To the best of our knowledge, no randomized clinical trials to date have investigated the effects of vegetarian or vegan diets on cognitive impairment or dementia outcomes. Our search of ongoing randomized clinical trials identified only one study testing the effects of a low-fat vegan diet on dementia (NCT04606420).

## Potential health risks of vegan and vegetarian diets

Accumulating evidence indicate that some vegetarians, especially vegans who are consuming restrictive diets, are at greater risk of developing haemorrhagic stroke, bone fractures, and a range of vitamin and mineral deficiencies that are particularly dangerous for growing children and pregnant and breastfeeding women.^136,137^ Vitamin B12, for example, is an essential vitamin produced by specific strains of soil bacteria that animals ingest when grazing grass. During digestion, large amounts of vitamin B12 are formed and incorporated in the animal’s meat, milk, and eggs. Fish and shellfish also contain considerable amount of vitamin B12; for instance, 100 g of clams contain up to 49 µg of vitamin B12. People following strict vegan diets must take a vitamin B12 supplement and/or consume foods supplemented with vitamin B12, including vitamin B12–fortified nutritional yeast, to avoid developing megaloblastic anaemia, a potentially irreversible form of neuropathy, and impaired bone formation. Vitamin B12 in spirulina or other algae is not bioavailable and may even inhibit vitamin B12 metabolism,^136^ but vitamin B12 in duckweed is bioavailable.^138^ Other potential deficiencies that vegetarians may develop are those from iron and zinc and occasionally riboflavin.^139^ These deficiencies are especially important in vegan children, pregnant/breastfeeding women, and those with menorrhagia. Many plant foods contain iron and zinc, but their bioavailability is limited due plant anti-nutrients, such as phytates, tannins, lectins, and oxalates. Cooking, sprouting, fermenting, and processing plant foods with vitamin C rich foods can increase iron and zinc absorption.^140^ Dietary calcium deficiency especially when coupled with protein restriction and excessive sodium intake can increase the risk of bone fractures in ethical vegans who do not consume healthy diets rich in calcium- and protein-rich plant foods.^93,137,141–143^ Many plants contain calcium, and in some of these, its bioavailability is very high. For instance, 40%–60% of the calcium contained in cabbage, broccoli, or broccoli sprouts is absorbed because of their low oxalate content, against only 31%–32% of the calcium in cow’s milk.^144^ Legumes, soy products (especially tofu made with calcium sulphate), and figs are also excellent sources of dietary calcium and protein. Regular exercise training, adequate sun exposure, and vitamin D supplementation are also important to promote bone health and prevent fractures^145^ and may play a key role in the protection against certain autoimmune diseases and advanced (metastatic) cancers.^146,147^

## The importance of consuming healthy vegetarian diets

Vegetarians should pay close attention to the quality and composition of their diets. Data from epidemiological studies suggest that men and women consuming plant-based diets rich in healthier plant foods (fresh vegetables, legumes, minimally processed whole grains, fruits, nuts, monounsaturated-rich vegetable oils, tea, and coffee) have lower risks of CHD and overall mortality with regular fish intake providing additionally health benefits.^4,87,148–150^ In contrast, people eating ‘unhealthy’ plant-based diets that emphasize refined grains, potatoes, high-sodium preserved vegetables, fried goods, sweets, juices, and sweetened beverages experienced higher risk of CHD and mortality.^4,5^ Similar results have been found for T2DM.^5^ Plant-based food products marketed as vegetarian and/or vegan can be rich in refined starch, added sugar, HFCS, salt, partially hydrogenated (*trans*) fat, and saturated fatty acids from tropical oils (e.g. one tablespoon of coconut oil contains 12 grams of saturated fat). Consumption of ultra-processed foods rich in sucrose and in HFCS, even if labelled as ‘vegetarian’ or ‘vegan’, promotes the development of insulin resistance, cardiometabolic syndrome, fatty liver disease, CVD, and cancer.^151,152^ High salt intake not only increases the risk of developing hypertension, CHD, and stroke,^55,153^ but it also triggers inflammation by increasing monocyte CCR2 expression.^154^  *Trans*-fatty acids from partially hydrogenated oils have markedly adverse effects on serum lipids, systemic inflammation, endothelial function, and ultimately on the risk of developing T2DM and CVD.^155^ However, naturally occurring *trans*-fatty acids found in milk and meat of ruminant animals have also similar adverse effects on LDL cholesterol, total cholesterol to HDL cholesterol ratio, and apolipoprotein B levels as do industrially produced *trans*-fatty acids.^156^ Finally, people consuming unhealthy vegetarian diets rich in refined carbohydrates might also be at risk of protein malnutrition. Plant foods contain all the nine essential amino acids but in different proportions. Legumes, for instance, are high in lysine, but low in tryptophan and methionine. In contrast, whole grains are low in lysine but high in tryptophan and methionine. Therefore, it is essential to consume every day a mixture of whole grains, beans and nuts, and/or protein-rich plant foods (e.g. tofu and mankai, a cultivated strain of the *Wolffia globosa* aquatic plant) to provide adequate amounts of all the essential and non-essential amino acids.

## Conclusions

Consuming vegetarian diets rich in minimally processed plant foods has been associated with a reduced risk of developing multiple chronic diseases including CVD, diabetes, hypertension, cancer, and dementia. Data from randomized clinic trials have confirmed a protective effect of vegetarian diets for the prevention of diabetes, hypercholesterolaemia, hypertension, and overweight, but to date, no data are available for acute coronary syndrome, heart failure, stroke, cognitive impairment, and dementia, and there are very limited data for cancer. However, since many individuals commonly and increasingly adopt vegetarian diets worldwide for ideological, cultural, environmental, and personal factors, it is of paramount importance to define which vegetarian dietary compositions provide better health outcomes and which components are detrimental to human health (*Graphical Abstract*).

New randomized trials are needed to understand whether the advantages of healthy plant-based diets represent an all-or-nothing phenomenon and if consuming less strict plant-based diets containing small quantities of animal products (e.g. pescatarian or traditional Mediterranean diets) has beneficial or detrimental effect on specific health outcomes, including the prevention of haemorrhagic stroke and bone fracture. Further, mechanistic studies are warranted to enhance our understanding about healthy plant-based food patterns and the biological mechanisms linking dietary factors and chronic diseases.

### Recommendations for clinicians and allied health practitioners

For overweight men and women seeking weight loss and cardiometabolic improvement as means of primary and secondary prevention of T2DM, hypertension, and CVD, well-balanced and supplemented vegetarian diets rich in minimally processed plant foods may be an option, especially when coupled with calorie restriction and regular exercise training as recommended in the 2018 Physical Activity Guidelines Advisory Committee Scientific Report.^28,157^ Regular fish intake can provide additional cardiovascular health benefits.^158^ Additional trials are warranted to determine whether patients with CVD will ultimately benefit from consuming vegetarian and vegan diets and, if so, in what ways. As with any potential therapeutic strategy, the risks and benefits of vegetarian diets must be discussed with patients. There is evidence to suggest that some vegetarians, particularly those who follow restrictive diets such as vegans, may be at greater risk of haemorrhagic stroke and bone fractures if they do not carefully plan their diets and consume fortified plant-based foods or supplements. In addition, vegans and some vegetarians may be at risk of deficiencies in vitamins and minerals such as vitamin B12, riboflavin, iron, zinc, calcium, and omega-3 fatty acids. This can be particularly dangerous for pregnant and breastfeeding women and growing children, as these nutrients are crucial for foetal and child development. It is recommended that anyone considering a vegetarian or vegan diet consult with a registered dietitian or healthcare provider to ensure that their diet is nutritionally adequate. Consuming vegetarian diets rich in refined grains, potatoes, high-sodium preserved vegetables, fried goods, sweets, juices, and sweetened beverages can increase the risk of developing T2DM and CVD morbidity and mortality. Finally, in the case of vegetarian diets and cancer, the benefits and risks are not well defined. As a weight loss strategy, this may be an option for some cancer patients, but there are currently no data to suggest that vegetarian or vegan diets in the absence of weight loss and/or changes in physical activity patterns will have a positive impact on cancer outcomes, including either recurrence or the development of metastatic cancers.

## Supplementary data

Supplementary data are not available at *European Heart Journal* online.

## Declarations

### Disclosure of Interest

All authors declare no conflict of interest for this contribution.

## Data Availability

Most extracted data and study materials are available from previously published research. Additional data extracted from the corresponding author of included studies will be shared upon reasonable request.
